# Lipopolysaccharide worsens the prognosis of experimental cerebral ischemia via interferon gamma-induced protein 10 recruit in the acute stage

**DOI:** 10.1186/s12868-019-0547-z

**Published:** 2019-12-27

**Authors:** Ping Wang, Jiaqi Zhang, Feifei Guo, Shuang Wang, Yi Zhang, Defeng Li, Haiyu Xu, Hongjun Yang

**Affiliations:** 1 Institute of Chinese Materia Medica, China Academy of Chinese Medical Sciences, Beijing, 100700 China; 2 Shaanxi Institute of International Trade & Commerce, Xianyang, 712046 China; 3 Heilongjiang University of Chinese Medicine, Harbin, 150040 China

**Keywords:** MCAO, LPS, Inflammation, Cerebral ischemia

## Abstract

**Background:**

Infection is an important clinical complication facing stroke-patients and triples the risk of death within 30 days post-stroke via mechanisms which are poorly understood.

**Aims:**

We tried to explore the mechanisms that inflammation caused by infections aggravated the ischemic brain injury after middle cerebral artery occlusion (MCAO).

**Methods:**

We used lipopolysaccharide (LPS) as systemic inflammatory stimuli to explore the mechanisms of aggravated ischemic brain injury after Sprague-Dawley male rats subjected to MCAO. Brain damage was evaluated by cerebral blood perfusion, Longa-5 scores, infarct volume and edema degree. Systemic cytokine responses and inflammatory changes in the plasma and brain were analyzed by ELISA kit, RT^2^ Profiler™ PCR array, and quantitative real-time PCR. The differential genes were subjected to Gene Ontology enrichment analysis and protein–protein interaction (PPI) network construction.

**Results:**

Lipopolysaccharide profoundly aggravated the brain damage after 24 h post-MCAO. At the acute stage (ischemia/reperfusion 90 min/3 h), the brain homogenate gene expression of interleukin 6 (IL-6), tumor necrosis factor α (TNF-α), interleukin 1β (IL-1β) and Interferon gamma-induced protein 10 (IP-10) was significantly up-regulated and the contents in plasma and brain homogenate were significantly increased in MCAO and MCAO + LPS group. IP-10 was the only gene with significant difference between MCAO and MCAO + LPS group, which was also in an important position with degrees of ≥ 14 in PPI network.

**Conclusions:**

It was possible that trace LPS aggravated the ischemic brain injury by induction of excessive IP-10 secretion in the acute stage, leading to excessive inflammatory response, which consequently increased the infarct volume and edema degree 24 h post-MCAO.

## Background

The incidence of first-ever stroke increased rapidly worldwide, and presented younger trend [1, 2]. Stroke is a devastating cerebrovascular event that the blood suddenly stops flowing smoothly to the brain, due to the blockage (ischemic stroke) or rupture (hemorrhagic stroke), which is a leading cause to morbidity and mortality. Numerous neuronal necrosis and immunocyte extreme infiltration is one of the hallmarks of ischemic stroke. Chemokines play an important role in inflammatory response [3]. Microglia, the resident macrophage population of the central nervous system (CNS) could be activated by any type of brain pathology and migrate to the injury site as up-regulating the expression of chemokine receptor, which aggravates the inflammation in the injured area. Otherwise, chemokine controls the peripheral white blood cells to enter the ventricle through the blood–brain barrier with increased permeability, release a variety of pro-inflammatory cytokines and promote inflammatory response. Moderate activation of microglia and appropriate infiltration of leukocyte are beneficial to the removal of cell debris from infarcted areas. However, if the activation and recruitment last too long, excessive inflammatory response would aggravate brain injury. It is undoubtedly that the inflammation cascade induced by stroke aggravates nerve injury, but one of the important clinical complications of post-stroke is infection.

Infection is a major clinical manifestation of stroke patients [4, 5]. It is reported that 23–65% of patients suffer from post-stroke infections, of which pneumonia and urinary tract infections are the most common [6–10], and pneumonia triples the risk of death within 30 days post-stroke [11]. Infection refers to the local tissue and systemic inflammatory response caused by the invasion of bacteria, viruses, fungi, parasites and other pathogens into the human body [4]. In normal case, inflammation is the body's defensive response which could promote tissue repair and functional normalization. However, inflammation induced by post-stroke infection seriously affects the prognosis of stroke, suggesting that peripheral inflammation signals can be transmitted to the central nervous system, which further aggravates neuroinflammation.

**Fig. 1:**
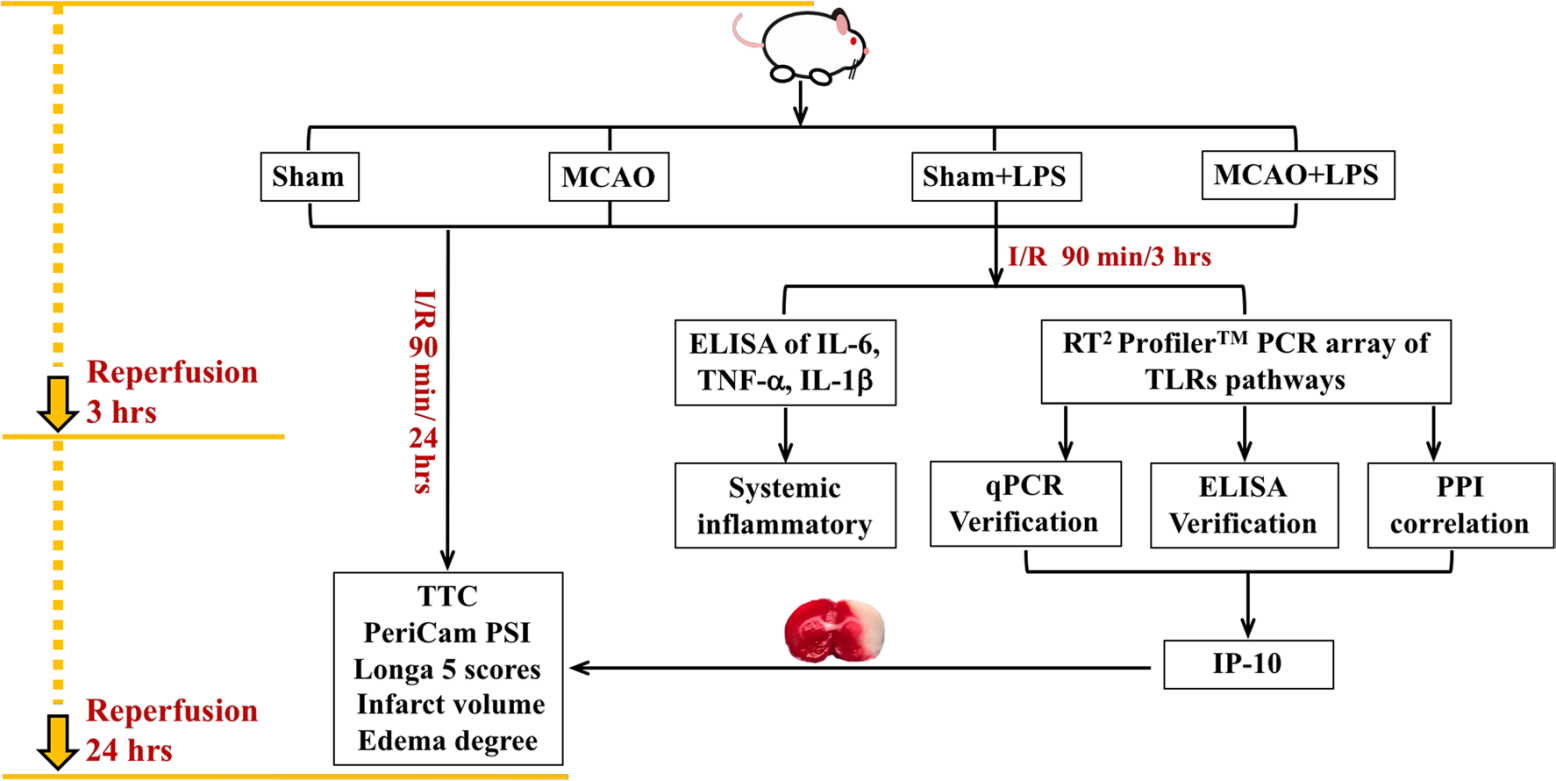
The flow chart of the experiment

Until now, there is no systematic report on how systemic inflammation caused by peripheral infection aggravates the prognosis of stroke. We speculate that infection-induced peripheral inflammation overlaps with stroke-induced central inflammation, and systemic inflammation aggravates the prognosis of stroke by aggravating related inflammation pathways. Nylon filament middle cerebral artery occlusion (MCAO) is the most popular method to simulate clinical cerebral ischemia, which was established by Koizum and Longa in 1980s [12, 13]. Lipopolysaccharide (LPS) is a component of the cell wall of Gram-negative bacteria, which is a classical agent for inducing inflammation [14–16]. Therefore, we employed LPS to induce peripheral inflammatory, rat middle cerebral artery occlusion (MCAO) to simulate clinical ischemic stroke, and the Toll-Like Receptor Signaling Pathway PCR Array to detect 84 genes known to be involved in the pathways to clarify the overlap key point of peripheral inflammation aggravating central inflammation in the acute stage of experimental cerebral ischemia, and to preliminarily reveal the target of alternative therapy for reducing stroke infection. The flow chart of the present experiment was shown in Fig. 1. It is possible that LPS-induced peripheral inflammation overlaps with stroke-induced central inflammation, and LPS aggravates the prognosis of stroke by aggravating related inflammation pathways, probably chemokine in the acute stage.

## Results

### Reduction in cerebral blood perfusion

**Fig. 2:**
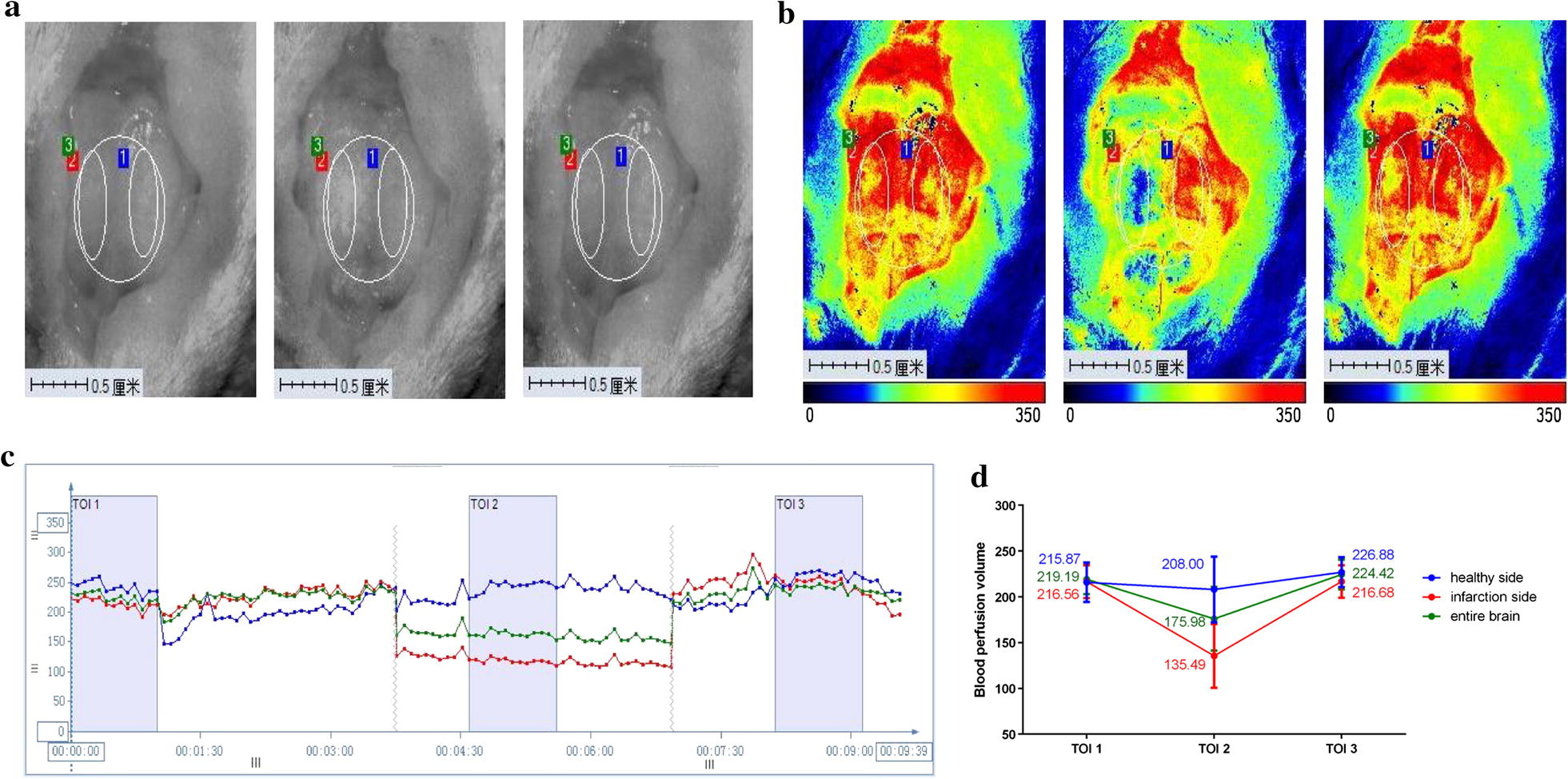
The results of PeriCam PSI monitoring (n = 6). **a** The axis top view of rat brain; **b** The CBP of SD rat before, during and after surgery; **c** The cerebral blood flow curve of SD rat before, during and after surgery; **d** The variation values of cerebral blood flow in SD rats. (ROI 1 delineated the healthy side, ROI 2 infarcted side and ROI 3 the whole brain, corresponding to blue line, red line and green line respectively in the blood flow curve. *TOI* time of interest)

The cerebral blood perfusion (CBP) was recorded before filament insertion, after filament insertion, and after filament pullout, at least 3 min each time. Figure 2a showed the axis top view of rat brain in chronological order. The skull showed slight white after filament insertion, suggesting ischemia in infarcted hemisphere. Figure 2b showed bright red and a little yellow in both hemispheres before filament insertion, indicating rich and smooth CBP in the whole brain. After filament insertion, infarcted hemisphere showed blue color, suggesting significantly decrease in CBP of MCA. Then the CBP of infarcted hemisphere basically restored to the preoperative level after filament pullout, with no difference between contralateral and ipsilateral hemisphere. Figure 2c showed the variation curves of bilateral hemisphere and the whole brain in chronological order, and cerebral blood flow decreased significantly in infarcted side (red line). The exact CBP values were shown in Fig. 2d and Table 1. Compared to the baseline, CBP of infarcted hemisphere was significantly decreased 37.57%, suggesting that the rat MCAO model was successfully replicated.

**Table 1:** The exact CBP values of bilateral hemisphere and the whole brain (n = 6)

Groups	Before surgery	After surgery	Filament pullout	Decrease rate of CBP %
Healthy hemisphere	215.87 ± 21.60	208.00 ± 35.93	226.88 ± 16.33	3.33 ± 5.98
Infarcted hemisphere	216.56 ± 17.92	135.49 ± 34.82**	216.68 ± 17.80^##^	37.57 ± 5.59
Whole brain	219.19 ± 16.25	175.98 ± 34.85**	224.41 ± 16.19^##^	19.69 ± 6.32

P < 0.05*, P < 0.01**, compared with before surgery; P < 0.05^#^, P < 0.01^##^, compared with after surgery

### System inflammation induced by LPS worsens outcome after MCAO surgery

**Fig. 3:**
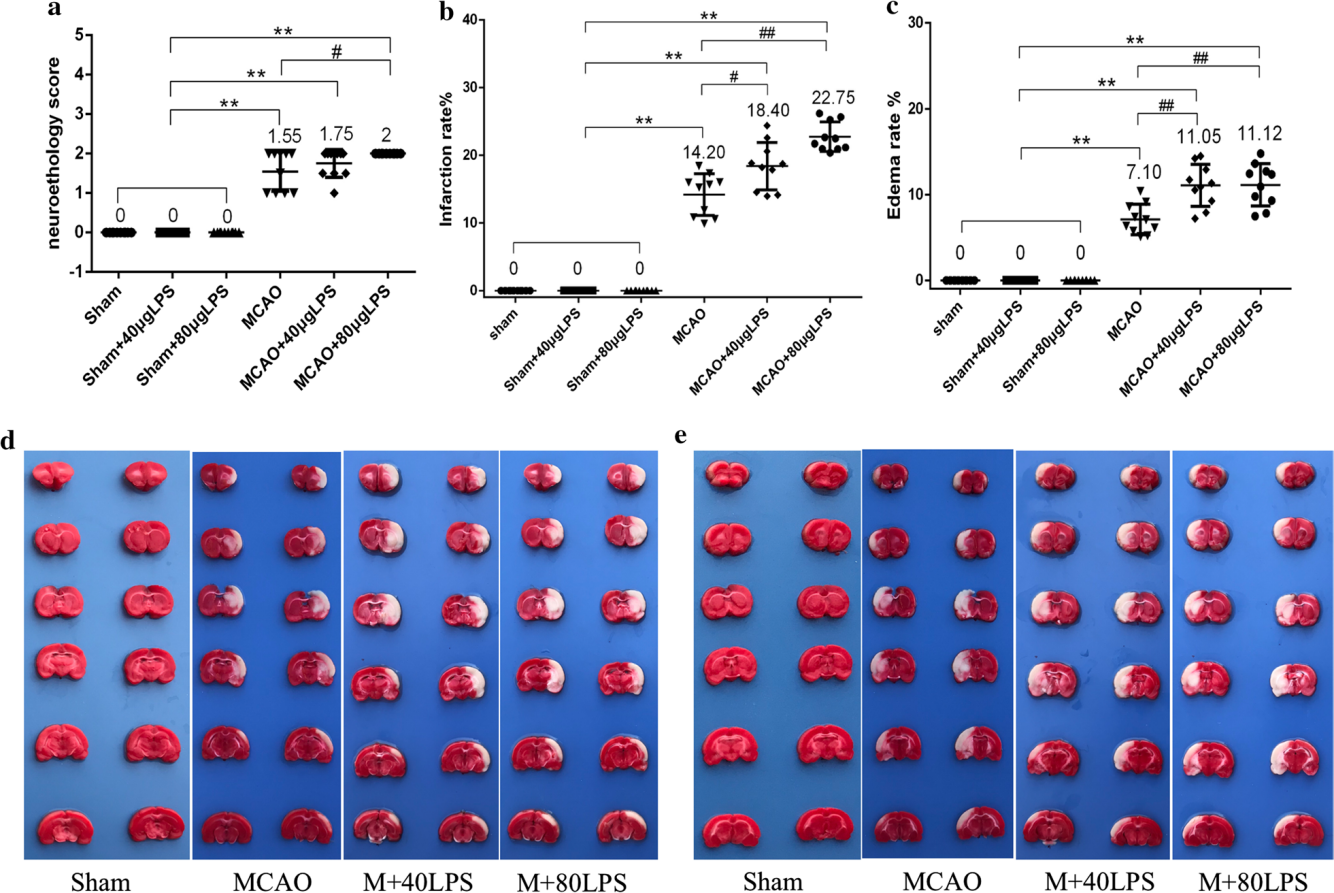
Systemic LPS exacerbates the extent of brain damage (n = 10). **a** LPS increased the neurological score; **b** LPS increased the infarct volume; **c** LPS increased the edema degree; **d** Infarction volume detected by TTC staining (the frontal sides); **e** Infarction volume detected by TTC staining (the reverse sides). (P < 0.05*, P < 0.01** compared with Sham group; P < 0.05^#^, P < 0.01^##^, compared with MCAO group.)

To determine the effect of a systemic inflammatory stimulus on cerebral ischemia/reperfusion injury, rats were subjected to LPS intraperitoneal injection at doses of 40 μg/ 300 g rat (134 μg/kg) or 80 μg/ 300 g rat (268 μg/kg) immediately after sham or MCAO surgery and the extent of brain damage was evaluated 24 h post-MCAO. No rats died during the whole procedure. The extent of brain damage was evaluated by neurological score, infarct volume and edema degree. 40 μg LPS caused 12.9%, 29.58% and 55.63% increase in neurological score, infarct volume and edema degree respectively compared with vehicle treatment. 80 μg LPS caused 29.0%, 60.21% and 56.62% increase in neurological score, infarct volume and edema degree respectively compared with vehicle treatment (Table 2 and Fig. 3). The aggravated injury was mostly attributable to exacerbation of cortical damage (Fig. 3d, e), and significantly increased the severity of neurological deficit. Moreover, the infarct site caused by 80 μg LPS was more uniform and thorough, but the edema degree was similar compared with that of 40 μg LPS. Therefore, 80 μg LPS was selected as the dosage in the following experiments.

**Table 2:** The extent of brain damage in each group (n = 10)

Groups	n	Neurological score	Infarct volume %	Edema degree %
Sham	10	0	0	0
Sham + 40 μg LPS	10	0	0	0
Sham + 80 μg LPS	10	0	0	0
MCAO	10	1.55 ± 0.50**	14.20 ± 3.09**	7.10 ± 1.77**
MCAO + 40 μg LPS	10	1.75 ± 0.35**	18.40 ± 3.52**^#^	11.05 ± 2.45**^##^
MCAO + 80 μg LPS	10	2.00 ± 0.00 **^#^	22.75 ± 2.17** ^##^	11.12 ± 2.46** ^##^

P < 0.05*, P < 0.01** compared with Sham group; P < 0.05^#^, P < 0.01^##^, compared with MCAO group

### System inflammation induced by LPS are dominated by different circulating cytokines

**Fig. 4:**
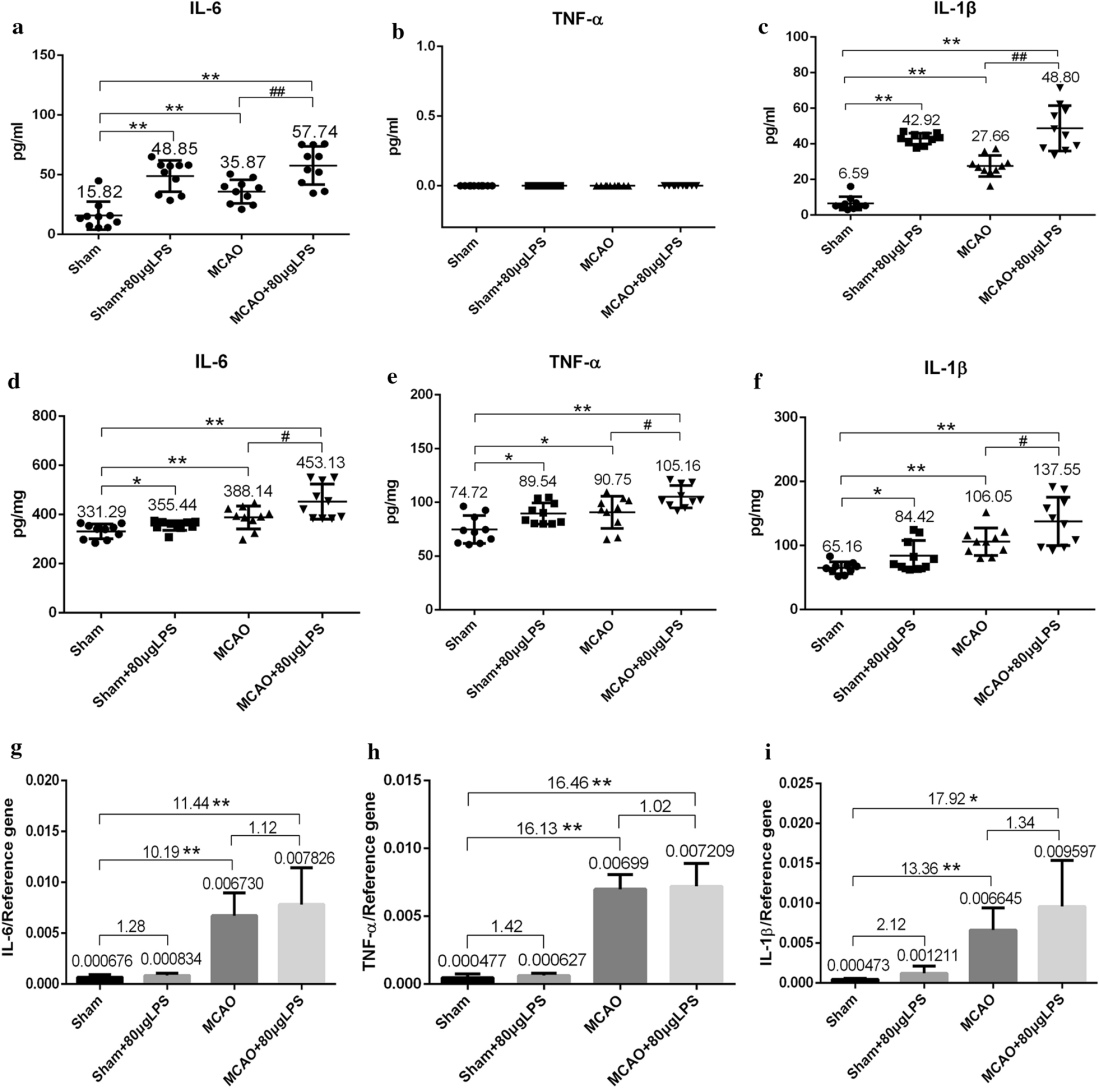
The levels of IL-6, TNF-α, IL-1β in the plasma and brain homogenates after systemic inflammatory challenges induced by LPS 4.5 h post-MCAO (n = 10). **a** IL-6 levels in plasma; **b** TNF-α levels in plasma; **c** IL-1β levels in plasma; **d** IL-6 levels in brain homogenates; **e** TNF-α levels in brain homogenates; **f** IL-1β levels in brain homogenates; **g** The mRNA expression of IL-6; **h** The mRNA expression of TNF-α; **i** The mRNA expression of IL-1β. (Three cytokines were measured by ELISA kit. Mean ± SEM values are shown. One-way ANOVA followed by Bonferroni’s comparison.)

To investigate the systemic inflammatory responses in actuate state, we investigated the plasma levels of three key cytokines 4.5 h post-MCAO, that was also after 4.5 h of LPS administration. LPS induced profound increases in IL-6 and IL-1β in both 80 μg LPS administrated groups. The level of IL-6 increased 3.00-fold in Sham + LPS group, 3.65-fold in MCAO + LPS group compared with that in Sham group (Fig. 4a). The level of IL-1β increased 6.51-fold in Sham + LPS group, 7.41-fold in MCAO + LPS group compared with that in Sham group (Fig. 4c). The systemic inflammatory responses caused by MCAO seemed not serious in the early stage (4.5 h), as the level of IL-6 and IL-1β was only 2.27-fold and 4.20-fold compared with that in Sham group. While no TNF-α was detected (Fig. 4b).

Simultaneously, we also investigated the brain homogenates levels of three key cytokines 4.5 h post-MCAO. MCAO induced profound increase in IL-6, TNF-α, IL-1β and LPS aggravated the local inflammation in the brain. The level of IL-6 increased 1.17-fold in MCAO group, 1.37-fold in MCAO + LPS group compared with that in Sham group (Fig. 4d). The level of TNF-α increased 1.21-fold in MCAO group, 1.41-fold in MCAO + LPS group compared with that in Sham group (Fig. 4e). The level of IL-1β increased 1.63-fold in MCAO group, 2.11-fold in MCAO + LPS group compared with that in Sham group (Fig. 4f). LPS alone also induced local inflammation in the brain to a small extent, as the levels of IL-6, TNF-α, IL-1β were 1.07-fold, 1.20-fold and 1.30-fold respectively compared with that in Sham group.

### Differential gene expression in experimental cerebral ischemia

**Fig. 5:**
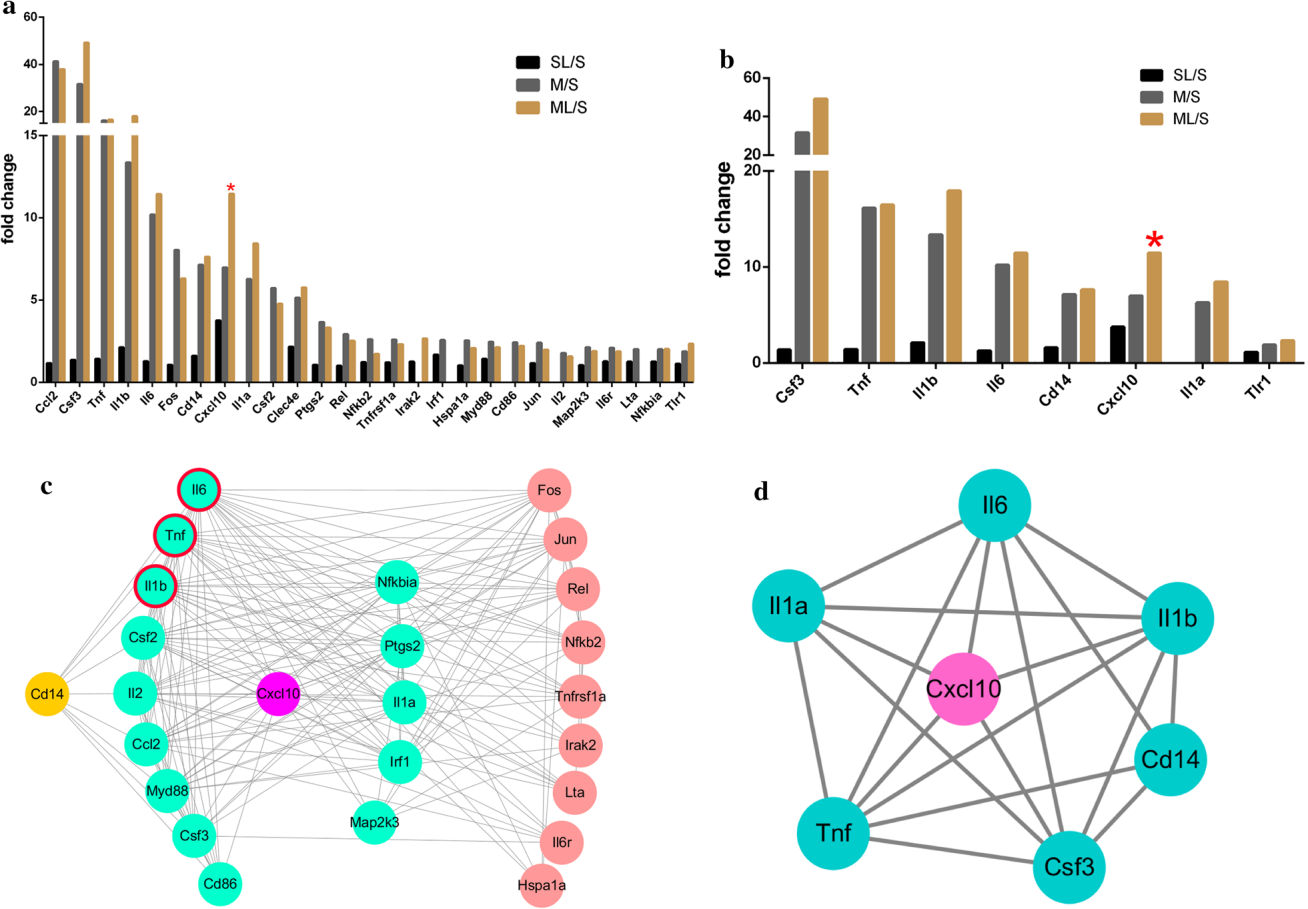
The fold changes and PPI network of different genes. **a** The fold changes of 27 different genes in different groups (n = 4). **b** The fold changes of 8 up-regulated genes in MCAO + LPS group compared with that in MCAO group (n = 4). **c** The PPI network of 27 different genes in MCAO or MCAO + LPS group compared with that in Sham group. **d** The PPI network of 8 up-regulated genes in MCAO + LPS group compared with that in MCAO group. (**c** The green nodes directly connected with Cxcl 10, and the green nodes in the left side also directly connected with Cd 14 which was associated with LPS function. The pink nodes indirectly connected with Cxcl 10; **d** Cxcl 10 directly connected with five nodes. S stands for Sham group, SL stands for Sham + LPS group; M stands for MCAO group, and ML stands for MCAO + LPS group.)

To get insight into the mechanism of more serious cerebral injury induced by LPS, we detected 84 genes involved in Toll-Like receptor signaling pathway 4.5 h post-MCAO. The data in details was shown in Table 3 and Fig. 5a. LPS alone didn’t caused significant changes after administrated to Sham group, as no genes had significant 1.5-fold changes. Twenty-five genes were up-regulated more than 1.5-fold after subjected to MCAO surgery and chemokine (C–C motif) ligand 2 (Ccl2) had the greatest changes in expression as 41.26-fold. Twenty-three genes had up-regulated more than 1.5-fold after subjected to MCAO + LPS and colony stimulating factor 3 (Csf3) had the greatest changes in expression as 49.21-fold. Interestingly, when we compared with MCAO and MCAO + LPS group, we found that 8 genes were up-regulated but only chemokine (C-X-C motif) ligand 10 (CXCL10) had significant 1.64-fold expression (Fig. 5b). The expression of IL-6, TNF-α, IL-1β were 1.28-, 1.42- and 2.12-fold changes respectively in Sham + LPS group compared with that in Sham group, but without significant differences. The expression of IL-6, TNF-α, IL-1β in MCAO or MCAO + LPS group was significantly up-regulated compared with that in Sham group (Fig. 4g–i).

**Table 3:** The fold change of 27 different genes between different groups 4.5 h-post MCAO (n = 4)

NO	Symbol	Gene name	SL/S		M/S		ML/S		ML/SL		ML/M	
			Fold Change	P value	Fold change	P value	Fold change	P value	Fold change	P value	Fold change	P value
1	Ccl2	Chemokine (C–C motif) ligand 2	1.16	0.624153	41.26	0.000511	37.94	0.000007	32.58	0.000007	− 1.09	0.533847
2	Csf3	Colony stimulating factor 3 (granulocyte)	1.36	0.424448	31.67	0.001058	49.21	0.003854	36.21	0.004366	2.61	0.277418
3	Tnf	Tumor necrosis factor (TNF superfamily, member 2)	1.42	0.397124	16.13	0.000025	16.46	0.000231	11.62	0.000251	1.02	0.835561
4	Il1b	Interleukin 1 beta	2.12	0.162953	13.36	0.004208	17.92	0.019435	8.45	0.028306	1.34	0.391086
5	Il6	Interleukin 6	1.28	0.400687	10.19	0.001754	11.44	0.007411	8.96	0.008197	1.12	0.623980
6	Fos	FBJ osteosarcoma oncogene	1.06	0.710817	8.04	0.002002	6.30	0.000222	5.93	0.000227	− 1.28	0.260185
7	Cd14	CD14 molecule	1.61	0.03763	7.14	0.005989	7.63	0.020957	4.73	0.029492	1.07	0.724771
8	Cxcl10	Chemokine (C-X-C motif) ligand 10	3.75	0.145277	6.98	0.002887	11.41	0.000000	3.04	0.010848	1.64	0.019928
9	Il1a	Interleukin 1 alpha	− 2.19	0.008363	6.27	0.000078	8.44	0.001116	18.50	0.000785	1.35	0.145179
10	Csf2	Colony stimulating factor 2 (granulocyte–macrophage)	− 2.06	0.25762	5.72	0.009292	4.77	0.056506	9.82	0.037527	− 1.20	0.920961
11	Clec4e	C-type lectin domain family 4, member e	2.16	0.156541	5.14	0.026359	5.76	0.000262	2.66	0.008226	1.12	0.964266
12	Ptgs2	Prostaglandin-endoperoxide synthase 2	1.06	0.629061	3.66	0.016385	3.32	0.016856	3.13	0.019496	− 1.10	0.719302
13	Rel	V-rel avian reticuloendotheliosis viral oncogene homolog	1.00	0.97117	2.92	0.000142	2.52	0.000300	2.51	0.000291	− 1.16	0.234818
14	Nfkb2	Nuclear factor of kappa light polypeptide gene enhancer in B-cells 2, p49/p100	1.22	0.032549	2.61	0.000035	1.72	0.001922	1.41	0.015419	− 1.51	0.004386
15	Tnfrsf1a	Tumor necrosis factor receptor superfamily, member 1a	1.20	0.163441	2.60	0.001290	2.31	0.000009	1.93	0.000025	− 1.13	0.270250
16	Irak2	Interleukin-1 receptor-associated kinase 2	1.25	0.528495	2.13	0.094605	2.66	0.019540	2.12	0.099720	1.25	0.502009
17	Irf1	Interferon regulatory factor 1	1.68	0.004013	2.57	0.001319	3.16	0.103964	1.88	0.205620	1.23	0.464775
18	Hspa1a	Heat shock 70kD protein 1A	1.03	0.831752	2.54	0.034229	2.08	0.029451	2.01	0.001303	− 1.22	0.266003
19	Myd88	Myeloid differentiation primary response gene 88	1.42	0.025778	2.46	0.001309	2.13	0.000289	1.49	0.000757	− 1.16	0.192018
20	Cd86	CD86 molecule	− 1.25	0.164326	2.42	0.003734	2.20	0.000224	2.74	0.000050	− 1.10	0.441172
21	Jun	Jun oncogene	1.16	0.391817	2.40	0.000204	1.98	0.006514	1.70	0.008321	− 1.21	0.130578
22	Il2	Interleukin 2	− 1.37	0.247581	1.78	0.246109	1.57	0.566071	2.15	0.015648	− 1.13	0.508809
23	Map2k3	Mitogen activated protein kinase kinase 3	1.05	0.670387	2.13	0.000237	1.89	0.000129	1.80	0.001076	− 1.12	0.112187
24	Il6r	Interleukin 6 receptor	1.27	0.145584	2.10	0.002435	1.88	0.007729	1.48	0.010756	− 1.12	0.392462
25	Lta	Lymphotoxin alpha (TNF superfamily, member 1)	1.25	0.264619	2.01	0.033772	1.16	0.520200	-1.07	0.993379	− 1.73	0.172841
26	Nfkbia	Nuclear factor of kappa light polypeptide gene enhancer in B-cells inhibitor, alpha	1.26	0.024038	2.00	0.000121	2.02	0.008320	1.60	0.021173	1.01	0.865093
27	Tlr1	Toll-like receptor 1	1.13	0.220799	1.87	0.031940	2.34	0.018029	2.08	0.024212	1.25	0.400292

S stands for Sham group, SL stands for Sham + LPS group; M stands for MCAO group, and ML stands for MCAO + LPS group

**Fig. 6:**
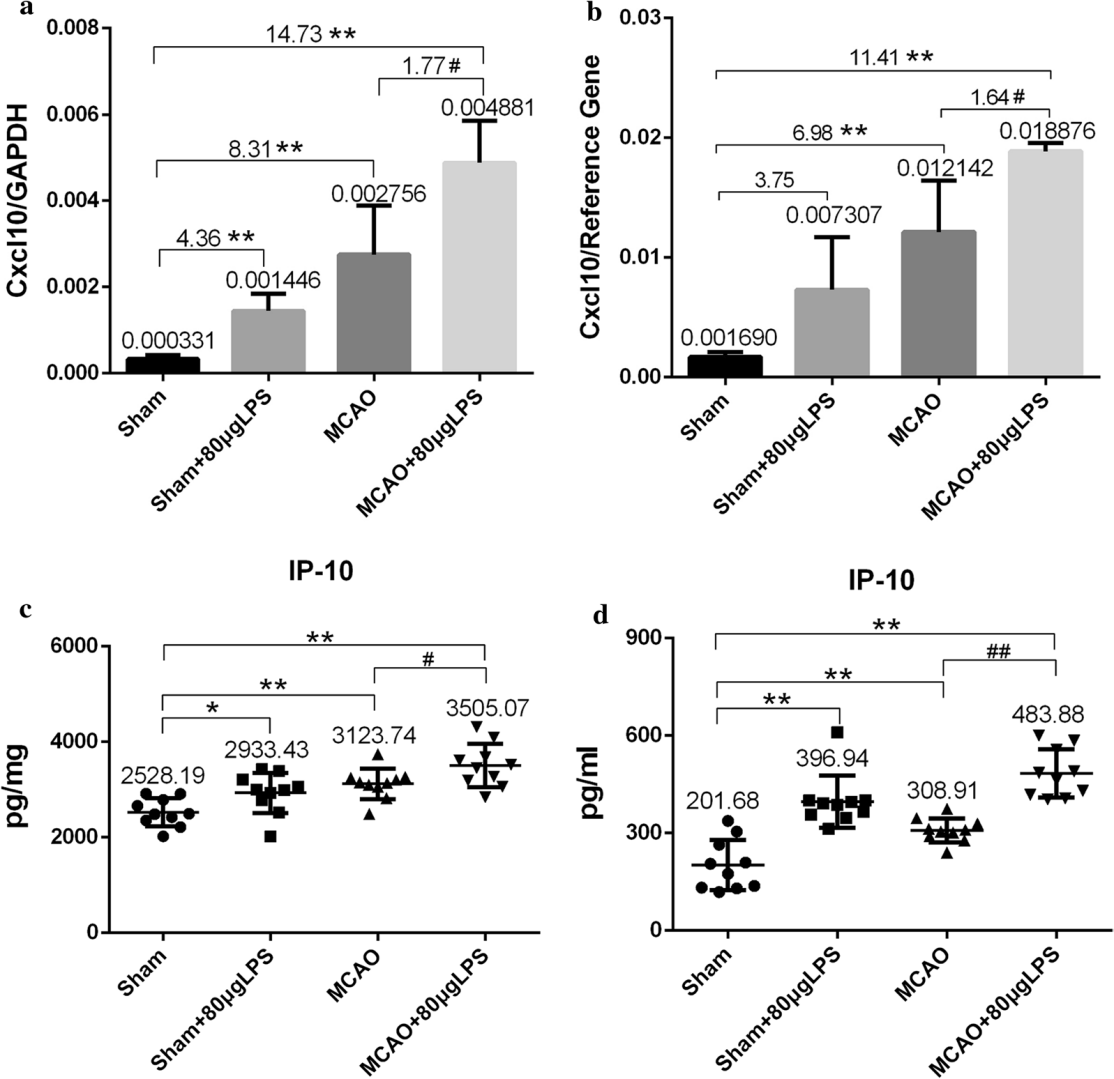
The mRNA level of Cxcl 10 and contents of IP-10 in different groups. **a** The mRNA level of Cxcl 10 detected by real-time PCR technique (n = 4). **b** The mRNA level of Cxcl 10 detected by PCR array respectively (n = 4). **c** The IP 10 levels in the brain homogenates measured by ELISA kit (n = 10). **d** The IP 10 levels in the plasma measured by ELISA kit (n = 10)

To confirm the expression of CXCL 10, we employed real-time PCR technique to quantitate its mRNA level. As shown in Fig. 6a, the mRNA level of CXCL 10 in Sham + LPS, MCAO and MCAO + LPS group was significant 4.36-fold, 8.31-fold and 14.73-fold changes respectively compared with that in Sham group. Moreover, LPS caused significant 1.77-fold change after subjected to MCAO surgery. The results corresponded to those of PCR array as shown in Fig. 6b. The expression of CXCL 10 in Sham + LPS, MCAO and MCAO + LPS group was 3.75-, 6.98- and 11.41-fold changes respectively compared with that in Sham group and 1.64-fold change between MCAO and MCAO + LPS group.

### GO enrichment analysis

To investigate the functional changes in the pathological course of MCAO and MCAO + LPS, the 27 different genes between MCAO vs Sham or MCAO + LPS vs Sham were mapped to DAVID database. This project provided three structured networks of defined terms to describe the gene product attributes: Biological process (BP), molecular function (MF) and cellular compartment (CC). The 27 different genes were up-regulated in the MCAO or MCAO + LPS group 4.5 h post-MCAO. The different genes were most commonly associated with BP, including immune response, inflammatory response, response to wounding and etc. (Table 4).

**Table 4:** The significantly enriched GO terms with a high count of different genes 4.5 h post-MCAO

Term	Category	Description	Count	P-value
GO:0006955	BP	Immune response	15	9.38E−15
GO:0006954	BP	Inflammatory response	12	7.31E−14
GO:0009611	BP	Response to wounding	12	8.33E−12
GO:0006952	BP	Defense response	12	1.30E−10
GO:0010604	BP	Positive regulation of macromolecule metabolic process	12	5.05E−09
GO:0045449	BP	Regulation of transcription	12	0.001056
GO:0010557	BP	Positive regulation of macromolecule biosynthetic process	11	1.44E−08
GO:0042127	BP	Regulation of cell proliferation	11	1.67E−08
GO:0031328	BP	Positive regulation of cellular biosynthetic process	11	2.13E−08
GO:0009891	BP	Positive regulation of biosynthetic process	11	2.32E−08
MF00016	MF	Signaling molecule	11	1.78E−07
GO:0001817	BP	Regulation of cytokine production	10	1.68E−12
GO:0005615	CC	Extracellular space	10	3.33E−08
GO:0010628	BP	Positive regulation of gene expression	10	1.13E−07
GO:0044421	CC	Extracellular region part	10	1.15E−06
GO:0005576	CC	Extracellular region	10	5.80E−04
GO:0006355	BP	Regulation of transcription, DNA-dependent	10	7.87E−04
GO:0051252	BP	Regulation of RNA metabolic process	10	8.82E−04
MF00017	MF	Cytokine	9	3.90E−12
GO:0045893	BP	Positive regulation of transcription, DNA-dependent	9	4.94E−07

*GO* Gene Ontology, *BP* biological process, *CC* cellular compartment, *MF* molecular function

### PPI network construction

The 27 different genes between MCAO vs Sham or MCAO + LPS vs Sham were imported to STRING database to construct the PPI network. Betweenness refers to the number of edges passing through the node. Closeness calculates the total distance to other nodes. The degree is the node number directly connecting with target node in the network. A higher value for the degree indicates a tight connected network and is likely to more robust. A total of 25 genes were screen with degree > 5, two genes (Clec4e and Tlr1) with degree 0, indicating both of them had no relationship with others (Table 5). Interesting, the degrees of IL-6, TNF-α, IL-1β were ≥ 21 and in the top three order, suggesting they may have an important role in MCAO and MCAO + LPS induced cerebral injury. The degree of CXCL 10 were 14, that was 14 genes were directly connecting with CXCL 10. The PPI network was shown in Fig. 4c, the green nodes represented the genes that directly connecting with CXCL 10, the others indirectly connecting. The green nodes in the left side were also directly connecting with Cd 14, which was closely related to the function of LPS.

**Table 5:** The details about PPI network of 27 different genes

No	Name	Degree	Betweenness centrality	Closeness centrality
1	Il6	24	0.09513658	1
2	Tnf	22	0.05768834	0.92307692
3	Il1b	21	0.04059896	0.88888889
4	Csf2	21	0.03921008	0.88888889
5	Il2	20	0.03323299	0.85714286
6	Ccl2	19	0.02477026	0.82758621
7	Nfkbia	18	0.0268796	0.8
8	Myd88	17	0.01976428	0.77419355
9	Fos	16	0.02109266	0.75
10	Ptgs2	16	0.01153843	0.75
11	Jun	15	0.015889	0.72727273
12	Il1a	14	0.01154667	0.70588235
13	Csf3	14	0.00856028	0.70588235
14	Cxcl10	14	0.01107399	0.70588235
15	Irf1	14	0.00318066	0.70588235
16	Rel	13	0.00255087	0.68571429
17	Nfkb2	12	0.00695873	0.66666667
18	Tnfrsf1a	11	0.00973399	0.64864865
19	Cd86	10	4.03E−04	0.63157895
20	Cd14	9	0	0.61538462
21	Irak2	7	0.00366201	0.58536585
22	Lta	7	4.53E−04	0.58536585
23	Map2k3	7	0.00535152	0.58536585
24	Il6r	6	0	0.57142857
25	Hspa1a	5	0	0.55813953
26	Clec4e	0	0	0
27	Tlr1	0	0	0

We also construct the PPI network of the eight genes which up-regulated in MCAO + LPS group compared with that MCAO group. Except for Tlr 1, the other seven genes were connected with each other. The degrees of IL-6, TNF-α, IL-1β were 6, and also in the top three order. CXCL 10 as the only significantly up-regulated gene was directly connecting with five genes (Fig. 4d and Table 6).

**Table 6:** The details about PPI network of eight different genes

No	Name	Degree	Betweenness centrality	Closeness centrality
1	Il6	6	0.03333333	1
2	Tnf	6	0.03333333	1
3	Il1b	6	0.03333333	1
4	Csf3	6	0.03333333	1
5	Cxcl10	5	0	0.85714286
6	Il1a	5	0	0.85714286
7	Cd14	4	0	0.75
8	Tlr1	0	0	0

### Increased levels of CXCL10 in brain homogenates and plasma

The production of CXCL10 in brain homogenates and plasma was measured by ELISA kit according to the manufactures protocol. The levels of CXCL 10 in three treated groups were significantly higher than that in Sham group, and highest in MCAO + LPS group as 3505.07 pg/mg and 483.88 pg/ml, no matter in brain homogenates or plasma (Fig. 6c, d). Moreover, the levels in brain homogenates were generally ten times higher than that in plasma.

## Discussion

In the present study, we firstly provide the evidence that LPS (134 and 268 μg/kg) aggravated the neurological score, cerebral infarction area and edema rate after rat experimental cerebral ischemia 24 h, and the damage caused by 268 μg/kg was more uniform and stable. Then we focused on the acute stage (I/R 90 min/3 h) to explore the stacking point of peripheral inflammation and central inflammation in order to find alternative therapeutic targets for stroke infection from the source. The plasma levels of inflammatory cytokines could reflect the systemic inflammatory response induced by LPS and the spread of central inflammation to the peripheral. IL-6 and IL-1β were both responded to LPS or MCAO, and the response induced by LPS was more profound, 3.00-fold and 6.51-fold respectively in Sham + LPS group compared with that in Sham group (Fig. 4a–c), suggesting LPS successfully induced system inflammatory. The peripheral inflammatory response (plasma levels of IL-6 and IL-1β) induced by MCAO was not seriously increased compared with that induced by LPS only, but it was responded much more profoundly in the brain homogenate (Fig. 4d–f), suggesting the inflammatory response induced by MCAO was concentrated in the cerebral ischemia parts at the acute stage. Plasma TNF-α had no response to LPS or MCAO stimulation in the acute stage, which was consistent to the report by Yousuf [17]. The LPS administration after MCAO surgery was both aggravated the central and peripheral inflammatory responses, although the dosage was only 268 μg/kg. The dosage of LPS used for inducing rat inflammation was usually milligram level per kilogram [18–22], which were much higher than the dosage we used. Trace LPS, as the last straw to crush the camel, could significantly aggravate ischemia brain injury post-MCAO, which explained the high mortality rate in stroke infection patients from the experimental level.

Systemic inflammatory response was successfully induced after 4.5 h LPS intraperitoneal injection and LPS aggravated brain damage after MCAO surgery. To get insight into the mechanism of more serious cerebral injury induced by LPS, we employed RT^2^ Profiler™ PCR array to detect the 84 genes expression of Toll-like Receptor Signaling Pathway in the infarcted hemisphere, high-throughput screening the specific genes of stroke infection in the acute stage in order to reveal the possible alternative target. The genes expression of both MCAO and MCAO + LPS group has changed significantly (Table 3 and Fig. 5a), and eight genes were up-regulated in MCAO + LPS group compared with that in MCAO group, but only CXCL 10 had significant higher expression (Fig. 5b). CXCL 10 was also in an important position with degrees of ≥ 14 in PPI network (Fig. 5c, d), corresponding to the report by Quan [23]. To confirm the expression of CXCL 10, we employed real-time PCR technique to quantitate its mRNA level (Fig. 6a, b) and ELISA kit to detect the increased contents of CXCL 10 in brain homogenates and plasma from the protein level (Fig. 6c, d).

CXCL10 also known as interferon gamma-induced protein 10 (IP-10), whose specific receptor is C-X-C chemokine receptor 3 (CXCR3). CXCL 10, as the name suggested, is chemotactic cytokine, belonging to α-chemokine family. Chemokine controls the attraction of leukocytes to tissues, which is essential for inflammation and the host response to infection. Chemokines are thought to provide the signals that convert the low-affinity, selectin-mediated interaction into the higher-affinity, integrin-mediated interaction that leads to extravasation of leukocytes [3]. Therefore, chemokines are necessary in the activation of immune cells and the transport of peripheral immune cells across the blood–brain barrier. Microglia are the resident macrophage population of the CNS which could be activated by any type of brain pathology and migrate to the injury site. The chemokine IP-10 is expressed in neurons responding to ischemic brain injury and is a signaling candidate for activating microglia and directing them to the lesion site. It was reported that CXCR3, the specific receptor for IP-10, up-regulated in microglia and controlled microglial migration [24]. It has been reported that IP-10/CXCR3 had an important part in the pathological process of stroke patients [25, 26] and experimental cerebral ischemia [27–31]. The mRNA and protein expression of IP-10/CXCR3 was increased in a time-dependent manner after permanent occlusion of the middle cerebral artery, suggesting IP-10/CXCR3 may be a potential novel therapeutic target in focal stroke [32, 33]. That's exactly what have happened. Chemokines and chemokine receptors, as a new target of stroke treatment, have been paid much more attention, and both CXC and CC chemokines as candidate drugs have been under research development [34]. At present, we just got the preliminary results that LPS worsened the prognosis of experimental cerebral ischemia via IP-10 recruit in the acute stage. Subsequently, we plan to design inhibitor experiment of CXCR3 in order to tamp the proinflammatory effect of IP-10. If we get the positive results, CXCR3 may be the possible target for both stroke and stroke-infection.

## Conclusions

Taken together, it was possible that trace LPS aggravated the ischemic brain injury by induction of excessive IP-10 secretion in the acute stage, leading to excessive inflammation in the brain tissue, which consequently increased the infarct volume and edema degree 24 h post-MCAO. Chemokine IP-10 may be a diagnostic or prognostic biomarker (significantly increased in plasma) in ischemic stroke-infection and its specific receptor CXCR3 may be the alternative targets for stroke-infection therapy in the near future.

## Methods

### Animals

All procedures were approved by the Medicine Ethics Review Committee for animal experiments of the China Academy of Chinese Medical Sciences, and all efforts were made to minimize suffering of rats. Sprague-Dawley rats (specific pathogen-free grade, Certificate No. 2010–0034), weighing 300 ± 20 g, 8 weeks old, were used for the study, which were purchased from Vital River Laboratory Animal Technology Co., Ltd. (Beijing, China). Male rats were used in the initial study because estrogen was known to protect against ischemic injury [35–37]. However, future studies with female rats will have to be conducted to assess potential sex-dependent effects on the inflammatory response after MCAO. The animal experiment was carried out in Clean Grade Animal Center of Institute of Chinese Materia Medica, China Academy of Chinese Medical Sciences. The rats were housed in a controlled environment (21 ± 1 °C temperature, 55 ± 10% relative humidity) with a 12/12-h light/dark cycle and free access to water and standard diet. The sample size was calculated by a power analysis and previous inflammation studies of the MCAO model [38–40]. The rats were allowed to acclimate for 7 days before the experiment. Sixty rats were randomly divided into six groups with ten in each one, that was Sham group, Sham + 40 μg LPS group, Sham + 80 μg LPS group, MCAO group, MCAO + 40 μg LPS group, and MCAO + 80 μg LPS group.

### Transient focal cerebral ischemia

Transient focal cerebral ischemia was induced using the intraluminal filament model of MCAO, which was firstly described by Koizumi [12] and Longa [13] in 1980s. Briefly, rats were anesthetized with 1.5–2.0% isoflurane (Beijing ZS Dichuang Technology Development Co., Ltd., Beijing, China) using respiratory anesthesia machine (ZS-MV, Beijing ZS Dichuang Technology Development Co., Ltd., Beijing, China), and fixed on homeothermic electric blanket (37 ± 0.5 °C) throughout surgery until coming around. Nylon filament (tip diameter 0.38 ± 0.02 mm, polylysine coated) was inserted into the right external carotid artery (ECA), and advanced through the internal carotid artery (ICA) until it obstructed the MCA. Reperfusion was performed after 90 min occlusion in anesthetic state. Sham surgery was performed exactly the same as above, but the filament was immediately withdrawn after reaching the origin of the MCA. Following MCAO, rats were placed in temperature-controlled (37 ± 1.0 °C) recovery cages for 2 h to prevent post-surgery hypothermia. The order in which rats from different groups were subjected to MCAO was randomized.

### Neurological function assessment

Rats were evaluated for neurologic deficits 24 h after reperfusion (ischemia 90 min reperfusion for 24 h, I/R 90 min/24 h) according to Longa 5 scores [2] by a fixed investigator who was blind to the groups. The scoring criteria are as follows: 0 = no deficit; 1 = failure to fully extend left forepaw, mild neurological deficit; 2 = circling to the left, moderate neurological deficit; 3 = falling to the left, severe neurological deficit; 4 = unable to walk spontaneously, conscious loss. This method is suitable for early stage of MCAO, within 7 days after surgery. The rats with 0 value in MCAO or MCAO + LPS group were eliminated and euthanized with intraperitoneal injection of 3% pentobarbital sodium salt (Sigma, USA) at 0.5 ml/100 g.

### Measurement of infarct volume and edema degree

Rats were anaesthetized with I.P. of 1% pentobarbital sodium salt. Brains were frozen on dry ice and serially sectioned into six coronal slices (2 mm) with brain mold. The brain slices were stained with 2% triphenyl tetrazolium chloride (TTC) at 37 °C for 15 min in the dark and fixed with 4% paraformaldehyde overnight. The infarct volume corrected for swelling, and edema degree were quantified using Image ProPlus Software by a fixed investigator who was blind to the groups, using the following formula [41–43]:

$${\text{Infarct rate\% }}=\frac{{\text{CoV } - \text{ IpV}}}{{{\text{2CoV}}}}{\text{100\%}};$$

$${\text{Edema rate\%}}=\frac{{\text{IpV + InV - CoV}}}{{{\text{2CoV}}}}{\text{100\%}};$$

**Fig. 7:**
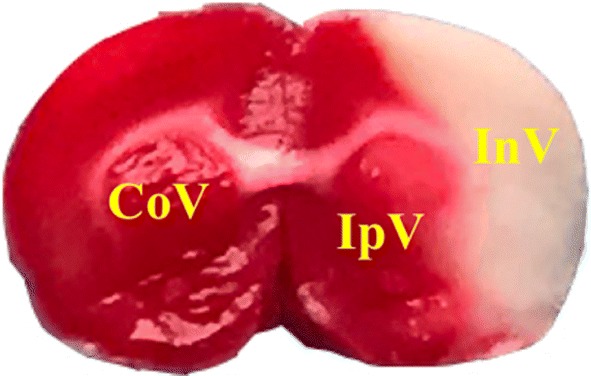
$${\text{Infarct rate\% = }}\frac{{\text{CoV } - \text{ IpV}}}{{{\text{2CoV}}}}{\text{100\%}};$$ $${\text{Edema rate\% = }}\frac{{\text{IpV + InV - CoV}}}{{{\text{2CoV}}}}{\text{100\%}}$$

CoV: contralateral hemisphere volume; IpV: ipsilateral no infarct volume; InV: infarct volume as shown in Fig. 7.

### Real-time cerebral blood perfusion

Cerebral blood perfusion (CBP) was dynamically and instantly monitored using Pericam Perfusion Speckle Image (PeriCam PSI) system by a fixed investigator who was blind to the groups, which could display the image and blood flow curve at the same time [44, 45]. The rats lied prone on an homeothermic electric blanket (37 ± 0.5 °C) under anesthesia. Head median incision was made to expose the entire parietal bone and scrape the skull with a scalpel. The laser spot is located 2 mm behind the anterior fontanelle and 6 mm beside the middle line. Keep the skull moist with 37 °C physiological saline throughout monitoring. Region of interest (ROI) 1 delineated the healthy side, ROI 2 infarcted side and ROI 3 the whole brain, corresponding to blue line, red line and green line respectively in the blood flow curve. Time of interest (TOI) delineated the relatively stable recording range to calculate the corresponding CBP, TOI 1 delineated the blood flow curve before surgery, TOI 2 during surgery and TOI 3 after surgery. CBP of two hemispheres and the whole brain was recorded before, during and after surgery at least 3 min each time.

$${\text{Reduced rate of CBP \% = }}\frac{{\text{T1IS } - \text{ T2IS}}}{{{\text{T1IS}}}}{\text{100\%}}.$$

T1IS: TOI 1 of infarcted side; T2IS: TOI 2 of infarcted side.

### Systemic inflammatory challenge with Lipopolysaccharide (LPS)

Lipopolysaccharide (LPS, serotype: 055: B5, Sigma L2880) was administered intraperitoneally at doses of 40 μg/ 300 g rat (134 μg/kg) or 80 μg/ 300 g rat (268 μg/kg) immediately after MCAO surgery [14–16]. No rats died or needed to be terminated due to LPS injection.

### Measurement of IL-6, TNF-α, IL-1β in plasma and brain homogenates by ELISA

5 ml blood was taken from inferior vena cava after rats subjected to I/R 90 min/3 h, then centrifuged at 3500 rpm, 4 °C for 10 min, and the plasma was stored in − 80 °C refrigerator for later use. The rat was decapitated and the infarcted hemisphere was rapidly freezed with liquid nitrogen and stored in − 80 °C refrigerator for later use. After balance to room temperature, the infarcted hemisphere was grinded with high throughput tissue lapping instrument (CK1000D, Thmorganh). 500 μl PMSF: RIPA (1:100) lysis buffer and 1 μl protease inhibitor was added to 100 mg rats brain homogenates. The mixture was re-grinded to thoroughly blended, and then centrifuged at 14000 rpm, 4 °C for 10 min, and the supernatant was used for determination of protein concentration by BCA Protein Assays kit (Thermo Fisher Scientific, USA) according to the manufactures protocol by a fixed investigator who was blind to the groups. Interleukin 6 (IL-6), tumor necrosis factor α (TNF-α), interleukin 1β (IL-1β) in plasma and brain homogenates was measured by ELISA kit (Invitrogen, Carlsbad, CA, USA) according to the manufactures protocol.

### RT^2^ Profiler™ PCR array

Sixteen rats were divided into four groups with four in each one, that was Sham group, Sham + 80 μg LPS group, MCAO group, and MCAO + 80 μg LPS group. The rat subjected to I/R 90 min/3 h was decapitated, then the infarcted hemisphere was rapidly washed with RNase-free water and loaded into RNase-free EP tubes, and freezed with liquid nitrogen. Investigator was required to operate the whole process quickly to avoid RNA enzyme contamination. We employed the Toll-Like Receptor Signaling Pathway PCR Array (QIAGEN, German, PARN-018Z) to detect 84 genes known to be involved in the pathway. RNA isolation, DNase treatment, and RNA clean-up were performed according to the manufacturer’s protocol (Qiagen, Hilden, Germany). The isolated RNA was reverse transcribed into cDNA using the RT2 First Strand Kit (Invitrogen, Carlsbad, CA, USA). PCR was performed using the RT2 SYBR Green qPCR Master Mix (Invitrogen, Carlsbad, CA, USA) on an ABI PRISM7700 instrument (Applied Biosystems, Foster City, CA). Data normalization (ΔC_t_) was based on correcting all C_t_ values for the average C’_t_ values of several stable expressed housekeeping genes present on the array containing the gene-specific primer sets. [ΔC_t1_ (group 1) = average C_t_ − average of HK genes’ C’_t_ for group 1 array; ΔC_t2_ (group 2) = average C_t_ − average of HK genes’ C’_t_ for group 2 array]. The fold change between two groups was expressed as 2^−(ΔCt1 − ΔCt2)^, that is 2^−ΔΔCt^ [46]. All the procedures were conducted by a fixed investigator who was blind to the groups. Every group had four biological repeats.

### Quantitative real-time polymerase chain reaction (qRT-PCR) analysis

The mRNA level of CXCL10 (IP-10) in the brain tissue was quantitated by real-time PCR. Total RNA was extracted manually from brain tissue using TRIZOL (Invitrogen, Carlsbad, CA, USA), then RNA was reverse-transcribed to cDNA using SuperScript. III Reverse Transcriptase (Invitrogen, Carlsbad, CA, USA). The primers for CXCL10 designed by software Primer 5.0 were as follows: 5′ AGCCAACCTTCCAGAAGCACCA 3′ (sense) and 5′ TCATGGAAGTCGATGCAGGTGC3′ (antisense); for GAPDH used as internal control were as follows: 5′ GCTCTCTGCTCCTCCCTGTTCTA3′ (sense) and 5′ TGGTAACCAGGCGTCCGATA3′ (antisense). The cycling programs were as follows: 95 °C for 10 min for 1 cycle, then 95 °C for 10 s, 60 °C for 60 s, and 95 °C for 15 s for 40 cycles. The quantitative real-time PCR was performed using ViiA 7 Real-time PCR System (Applied Biosystems, Carlsbad, CA, USA) with 2× PCR master mix (Arraystar, USA) according to the manufacturer’s protocol. The gene CXCL10 concentration of each sample is corrected by its housekeeping gene GAPDH. Relative quantification was processed by the standard curve method. All the procedures were conducted by a fixed investigator who was blind to the groups.

### Measurement of CXCL10 production in brain homogenates and plasma by ELISA

The production of CXCL10 in brain homogenates and plasma was measured by ELISA kit (Cusabio biotech co., Ltd, WuHan, China) according to the manufactures protocol by a fixed investigator who was blind to the groups.

### GO enrichment analysis

To explore the gene function of the different genes, we employ the GO analysis for functional annotation. The 27 different genes were imported into DAVID Bioinformatics Resources 6.7 (https://david-d.ncifcrf.gov/), which supply a high-throughput and integrated data-mining environment. The results were downloaded from the internet.

### PPI network construction

To explore the protein–protein interaction (PPI) correlation, the different expression genes were mapped to the Search Tool for the Retrieval of Interacting Genes (STRING, https://string-db.org/) database. In the process of analysis, species were limited to "Rattus norvegicus" and the minimum interaction threshold was set to "medium confidence" 0.4. The other parameters were set by default, and the target with weak correlation was removed. Based on the above results of PPI analysis, Cytoscape v3.6.1 software was employed to describe the interaction relationship. The Network Analyzer was used to analyze the topological properties, and the targets with degree ≥ twofold Median were selected to construct the PPI network graph.

### Data analysis

Data were analyzed using Student’s *t* test for single comparisons and one-way ANOVA followed by Student’s *t* test with Bonferroni’s correction or Dunnett’s test for multiple comparisons. The criterion for statistical significance was p < 0.05. Data were expressed as mean ± standard error of the mean (SEM).

## Data Availability

The datasets used and/or analysed during the current study are available from the corresponding author on reasonable request.

## Abbreviations

BP: biological process (BP)
CC: cellular compartment
CNS: central nervous system
CBP: cerebral blood perfusion
Ccl2: chemokine (C–C motif) ligand 2
Csf3: colony stimulating factor 3
CXCL10: chemokine (C-X-C motif) ligand 10
CXCR3: C-X-C chemokine receptor 3
GO: Gene Ontology
IP-10: interferon gamma-induced protein 10
IL-1β: interleukin 1β
IL-6: interleukin 6
LPS: lipopolysaccharide
MCAO: middle cerebral artery occlusion
MF: molecular function
PeriCam PSI: Pericam Perfusion Speckle Image
PPI: protein–protein interaction
ROI: region of interest
TTC: triphenyl tetrazolium chloride
TNF-α: tumor necrosis factor α
